# The Algal Meroterpene 11-Hydroxy-1′-*O*-Methylamentadione Ameloriates Dextran Sulfate Sodium-Induced Colitis in Mice

**DOI:** 10.3390/md14080149

**Published:** 2016-08-05

**Authors:** Hanaa Zbakh, Elena Talero, Javier Avila, Antonio Alcaide, Carolina de los Reyes, Eva Zubía, Virginia Motilva

**Affiliations:** 1 Department of Pharmacology, Faculty of Pharmacy, University of Seville, Seville 41012, Spain; zbakh.h@hotmail.com (H.Z.); etalero@us.es (E.T.); javila@us.es (J.A.); antonioalcaide@hotmail.es (A.A.); 2 Department of Biology, Faculty of Sciences, University Abdelmalek Essaadi, Tetouan 93030, Morocco; 3 Department of Organic Chemistry, Faculty of Marine and Environmental Sciences, University of Cádiz, Puerto Real (Cádiz) 11510, Spain; carolina.dereyes@uca.es (C.d.l.R.); eva.zubia@uca.es (E.Z.)

**Keywords:** marine meroterpenoids, macroalgae, intestinal inflammation, experimental colitis, cytokines, COX-2, iNOS

## Abstract

Inflammatory bowel disease (IBD) is a complex class of immune disorders. Unfortunately, a treatment for total remission has not yet been found, while the use of natural product-based therapies has emerged as a promising intervention. The present study was aimed to investigate the anti-inflammatory effects of the algal meroterpene 11-hydroxy-1′-*O*-methylamentadione (AMT-E) in a murine model of dextran sodium sulphate (DSS)-induced colitis. AMT-E was orally administered daily (1, 10, and 20 mg/kg animal) to DSS treated mice (3% *w*/*v*) for 7 days. AMT-E prevented body weight loss and colon shortening and effectively attenuated the extent of the colonic damage. Similarly, AMT-E increased mucus production and reduced myeloperoxidase activity (marker for anti-inflammatory activity). Moreover, the algal meroterpene decreased the tumor necrosis factor (TNF)-α, interleukin (IL)-1β, and IL-10 levels, and caused a significant reduction of the expression of inducible nitric oxide synthase (iNOS) and cyclooxygenase-2 (COX-2). Our results demonstrate the protective effects of AMT-E on experimental colitis, provide an insight of the underlying mechanisms of this compound, and suggest that this class of marine natural products might be an interesting candidate for further studies on the prevention/treatment of IBD.

## 1. Introduction

Ulcerative colitis (UC) and Crohn’s disease (CD) are two typical forms of inflammatory intestinal disease belonging to inflammatory bowel disease (IBD) [1], which affect millions of people worldwide and carry a widespread health hazard in modern society [2]. IBD is characterized by chronic and recurrent inflammatory disorders of the gastrointestinal tract [3]. Despite many years of extensive research, the aetiology and pathogenesis of IBD is not completely understood. It probably involves a complex interaction of several factors, including genetic susceptibility, immune disorders, bacterial flora within the intestinal environment, and environmental factors [4]. Therefore, the development of new, effective, and well-tolerated drugs for IBD therapy is necessary. Various models of experimental IBD have been developed to investigate the pathogenesis of this disease and may be used to test innovative approaches for therapy [5]. Among these models, experimental colitis induced by dextran sulphate sodium (DSS) has been widely used because it affords a high degree of uniformity and reproducibility of most lesions in the distal colon, and after 7 days of DSS administration the intestinal immune system is activated to generate, in a coordinated form, pro-inflammatory/anti-inflammatory cytokines [6]. The pro-inflammatory cytokines amplify the inflammatory response by activating a cascade of immune cells such as neutrophils and macrophages. Infiltration of neutrophils results in the production of large amounts of cytotoxic reactive oxygen species, nitrogen metabolites, and lytic enzymes, which lead to severe inflammatory tissue injury, including mucosal disruption and ulceration [7].

**Figure 1:**
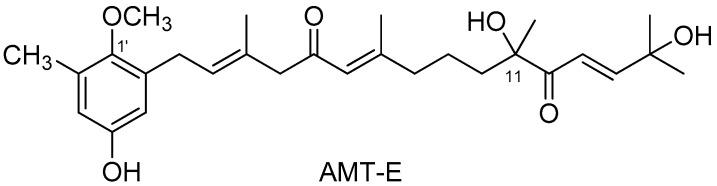
Chemical structure of the meroditerpene 11-hydroxy-1′-*O*-methylamentadione (AMT-E).

Macroalgae produce a wide variety of bioactive compounds that offer great opportunities in the biomedical field [8,9,10]. Although most research on the pharmacological potential of algal natural products has focused on cytotoxic and antimicrobial activities [8,9,11], recent studies are also disclosing interesting properties of algae-derived extracts and natural products as anti-inflammatory agents [12,13,14,15]. Thus, we have recently described the in vitro anti-inflammatory activity of a series of meroterpenoids isolated from the brown alga *Cystoseira usneoides* [16,17]. In particular, the meroditerpene 11-hydroxy-1′-*O*-methylamentadione (AMT-E) (Figure 1), which is one of the major natural products of this alga, showed significant activity as an inhibitor of the production of the pro-inflammatory cytokine TNF-α in LPS-stimulated THP-1 human macrophages [16].

The promising in vitro activity exhibited by AMT-E led us to investigate the potential in vivo anti-inflammatory effect of this natural product on the experimental colitis induced by DSS. We have found that AMT-E exerts intestinal anti-inflammatory activity in the colitis by increasing mucus production, inhibiting neutrophil infiltration, down-regulating TNF-α, IL-1β, and IL-10, as well as suppressing COX-2 and iNOS expression in the mouse colon tissue. 

## 2. Results

### 2.1. AMT-E Treatment Protects Mice against DSS-Induced Acute Colitis

**Figure 2:**
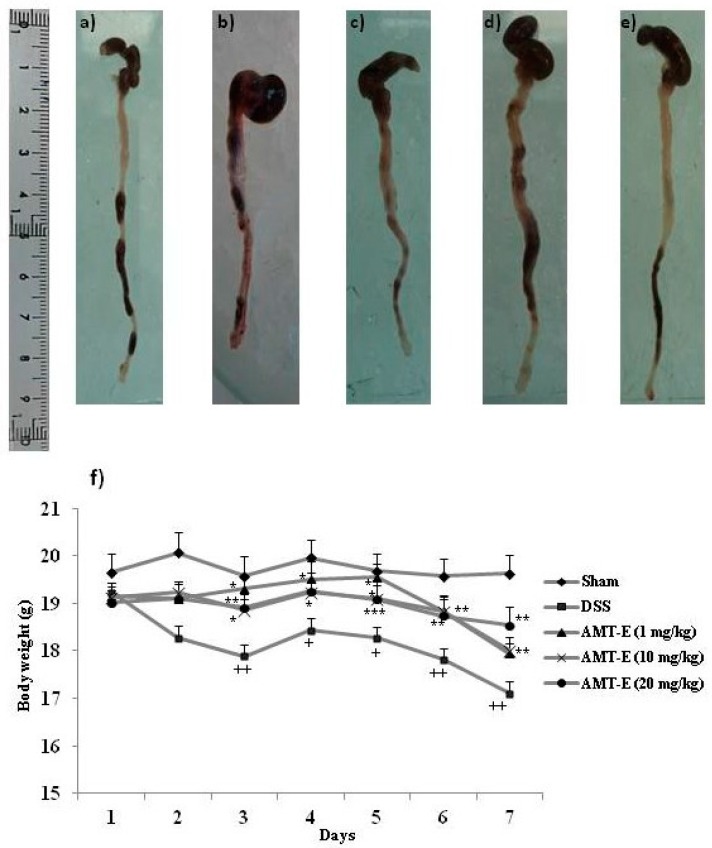
11-hydroxy-1′-*O*-methylamentadione (AMT-E) protect mice against dextran sodium sulphate (DSS)-induced colitis. (**a**–**e**) Representative macroscopic appearance of the colon in (**a**) Sham group (**b**) mice treated with DSS and (**c**–**e**) mice receiving DSS plus AMT-E (1, 10, and 20 mg/kg p.o., respectively); (**f**) Change in bodyweight during the study. Data are expressed as the means ± SEM. Statistical significance between Sham and DSS groups was determined by Student’s *t* test. The statistical differences between DSS and treatments groups were determined by oneway ANOVA followed by Bonferroni *post-hoc* test. ^+^ *p* < 0.05 and ^++^ *p* < 0.01 vs. Sham group; * *p* < 0.05, ** *p* < 0.01 and *** *p* < 0.001 vs. DSS group.

To determine the potential in vivo anti-inflammatory effect of AMT-E, we tested this compound using the DSS model of acute intestinal inflammation. It is worth noting that neither DSS nor the different AMT-E treatments caused changes in the relative weights and appearance of organs such as the liver, kidneys, and heart, which are highly susceptible to drug toxicity. However, as shown in Table 1, mice that were treated with DSS showed a marked colon shortening in relation to Sham animals (*p* < 0.001) (Table 1 and Figure 2a,b). This effect is indicative of the presence of inflammation. AMT-E treatments modified this effect and the highest dose of the compound (20 mg/kg) was able to significantly prevent DSS-induced colon shortening (*p* < 0.01) (Table 1 and Figure 2e). Moreover, mice that were treated with DSS showed a progressive loss of body weight, with fluctuations typical of this type of experiment, during the 7 days that the study lasted (Figure 2f). Mice treated with AMT-E were protected from this marked loss of body weight, with significant response from day three to the end, and for the three doses tested.

### 2.2. AMT-E Alleviates Microscopic Colon Damage and Increases Mucus Production

**Figure 3:**
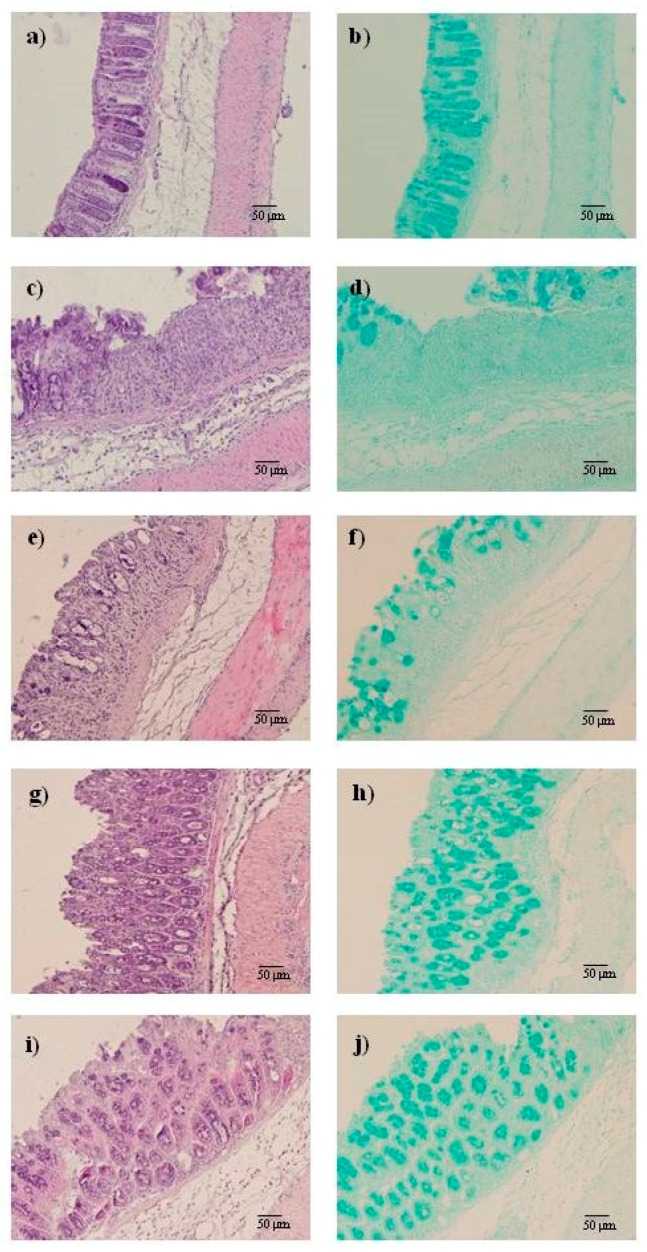
11-hydroxy-1′-*O*-methylamentadione (AMT-E) administration attenuate microscopic colon damage induced by dextran sulfate sodium (DSS); this effect is detected in Haematoxylin/Eosin and also in Alcian Blue stain sections where positive goblet cells were more clearly observed in preserved regions of the mucosal layer in mice treated with different dose of AMT-E. Histological appearance of samples taken from middle to distal colon of mice: (**a**,**b**) Sham group; (**c**,**d**) DSS group; (**e**,**f**) DSS plus AMT-E (1 mg/kg, p.o.); (**g**,**h**) DSS plus AMT-E (10 mg/kg, p.o.) and (**i**,**j**) DSS plus AMT-E (20 mg/kg, p.o.). Original magnification 200×.

Histological examination and results from the histopathological score for all groups are shown in Figure 3 and Table 2. Colons from Sham mice revealed typical features of normal structure (Figure 3a). Consistent with the macroscopic changes, where DSS-treated mice showed inflammation in the medial-distal area of the colon, this was also evident after the microscopic analysis involving all layers of the bowel wall (score 3.93 ± 0.2, Table 2); extensive granulation tissue with the presence of a massive neutrophilic infiltration, fibroblasts and lymphocytes was also apparent, mainly in the mucosa and submucosa; necrosis of epithelium, distortion of crypts, and partial destruction of the glands were also detected (Figure 3c); Alcian blue staining, which detects acid mucin-positive goblet cells, revealed remarkable mucin depletion in ulcerative areas of DSS-treated animals (Figure 3d) compared with Sham mice (Figure 3b). By contrast, the histological sections of the AMT-E-treated animals (Figure 3e,g,i) showed an improvement in the microscopic features of colitis at all the doses used, evidenced by a preservation of the colonic mucosa structure and a reduction of inflammatory cells in the lamina propria when compared to DSS group. Moreover, Alcian blue-positive goblet cells were clearly observed in preserved regions of the mucosal layer in mice treated (Figure 3f,h,j). This analysis provides a score of 2.34 ± 0.1, 2.12 ± 0.2, and 1.82 ± 0.2 for AMT-E doses of 1, 10, and 20 mg/kg respectively (*p* < 0.05 vs. DSS group; Table 2).

### 2.3. AMT-E Attenuates MPO Levels in DSS-Induced Colitis in Mice

**Figure 4:**
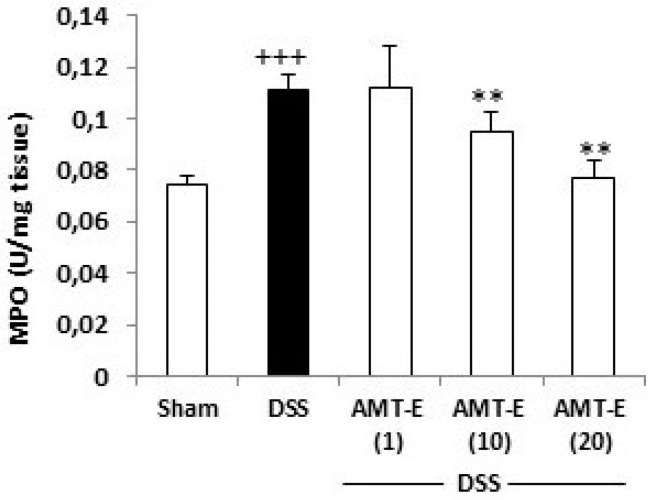
11-hydroxy-1′-*O*-methylamentadione (AMT-E) administration reduces leukocyte infiltration. Myeloperoxidase activity (MPO, U/mg tissue) was quantified in mice treated with DSS alone or mice receiving DSS plus AMT-E (1, 10, and 20 mg/kg, p.o.). The Sham group received vehicle in an equal volume. Data are expressed as the means ± SEM. Statistical significance between Sham and DSS groups was determined by Student’s *t* test. The statistical differences between DSS and AMT-E treated groups were determined by oneway ANOVA followed by Bonferroni post-hoc test. ^+++^ *p* < 0.001 vs. Sham group. ** *p* < 0.01 vs. DSS group.

The histopathology study indicated that a mechanism underlying the protective effects of AMT-E involved a reduced infiltration of inflammatory cells into the colonic mucosa. Thus, colon inflammation was quantitatively evaluated by MPO activity. As shown in Figure 4, DSS induced a significant increase in colon MPO activity from 0.07 ± 0.003, a basal concentration, to 0.11 ± 0.006 U/mg tissue (*p* < 0.001 vs. Sham group) after 7 days of DSS administration. The treatment with AMT-E at the doses of 10 and 20 mg/kg significantly suppressed the degree of polymorphonuclear neutrophil infiltration in colon tissue (0.09 ± 0.007 and 0.07 ± 0.0006 U/mg tissue, respectively, *p* < 0.01 vs. DSS group), Figure 4.

### 2.4. Effects of AMT-E on the Production of Cytokines in the Colon of DSS-Treated Mice

**Figure 5:**
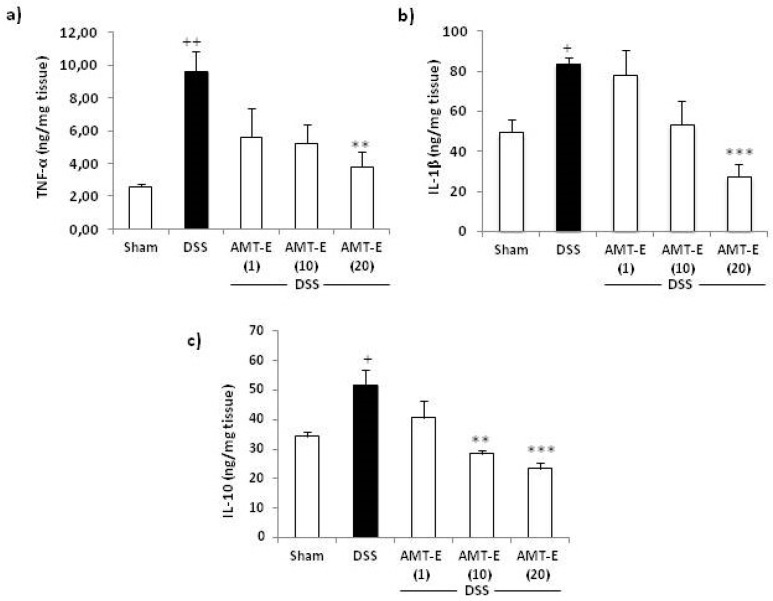
Effects of 11-hydroxy-1′-*O*-methylamentadione (AMT-E) administration on cytokine levels of colonic tissue in DSS-induced colitis. (**a**) TNF-α levels (ng/mg tissue); (**b**) IL-β levels (ng/mg tissue); and (**c**) IL-10 levels (ng/mg tissue), were quantified in mice treated with DSS alone and in those receiving DSS plus AMT-E (1, 10, and 20 mg/kg, p.o.). The Sham group received vehicle in an equal volume. Data are expressed as the means ± SEM. Statistical significance between Sham and DSS groups was determined by Student’s *t* test. The statistical differences between DSS and AMT-E treated groups were determined by oneway ANOVA followed by Bonferroni post-hoc test. ^+^ *p* < 0.05 and ^++^ *p* < 0.01 vs. Sham. ** *p* < 0.01 and *** *p* < 0.001 vs. DSS group.

To investigate the influence of AMT-E on cytokines production, we measured the levels of pro-inflammatory (TNF-α and IL-1β) and anti-inflammatory (IL-10) cytokines in the colonic tissue from different treatment groups. As shown in Figure 5, the levels of TNF-α and IL-1β were increased in the colon tissues from DSS-treated mice compared to that in control animals. Furthermore, the administration of AMT-E at a dose of 20 mg/kg significantly reduced the levels of these cytokines by 60% (*p* < 0.01) and 67% (*p* < 0.001), respectively, with respect to DSS group (Figure 5a,b). As regards IL-10, this cytokine was up-regulated in DSS-treated mice compared with Sham group. Interestingly, 10 and 20 mg/kg of AMT-E resulted in a significant decrease of IL-10 values compared with DSS group (Figure 5c).

### 2.5. AMT-E Downregulates the Expression of COX-2 and iNOS in Colonic Mucosa

**Figure 6:**
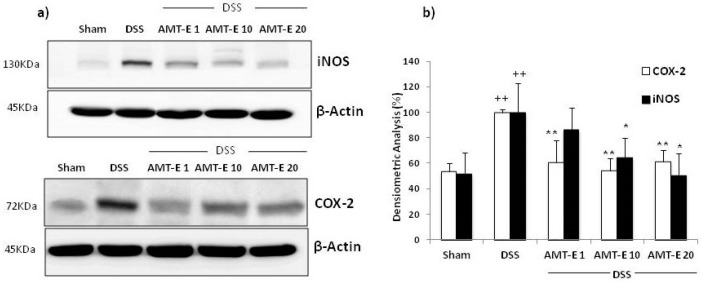
11-hydroxy-1′-*O*-methylamentadione (AMT-E) administration reduce colonic protein levels of COX-2 and iNOS enzymes in DSS-induced colitis. (**a**) Representative western blot analysis of COX-2 (*n* = 5) and iNOS (*n* = 4) proteins; (**b**) Densitometric data were studied following normalization to the control (housekeeping gene, β-Actin). Data are expressed as the means ± SEM. Statistical significance between Sham and DSS groups was determined by Student’s *t* test. The statistical differences between DSS and AMT-E treated groups were determined by oneway ANOVA followed by Bonferroni *post-hoc* test. ^++^ *p* < 0.01 vs. Sham group. * *p* < 0.05 and ** *p* < 0.01 vs. DSS group.

The effect of AMT-E on the expression levels of COX-2 and iNOS in cytosolic extracts from colonic mucosa was evaluated by Western blot analysis. The expression of both proteins was significantly increased in the colon tissues from DSS-treated mice compared with those in control mice (*p* < 0.01). However, administration of AMT-E significantly reduced the DSS-induced expression of COX-2 and iNOS that were decreased to basal levels with different doses of AMT-E (Figure 6).

## 3. Discussion

IBD is a group of inflammatory conditions of the gastrointestinal tract with the two major types including ulcerative colitis (UC) and Crohn’s disease (CD). The standard therapy for IBD includes mainly immunomodulatory agents, although their use entails severe side effects such as hormonal disturbance, peptic ulcer, liver dysfunction, and psychological problems [18]. Therefore, the need for alternative therapeutic approaches is an emerging strategy.

Algae have been used in traditional medicine to treat a variety of diseases [19], including gastrointestinal disorders such as vomiting, haemorrhoids, and dyspepsia [20]. Further, the protective effects of the alga *Sargassum pallidum* [21] and of the extracts of the alga *Laminaria japonica* [20] on IBD have recently been reported. In this context, the in vitro anti-inflammatory activity detected for the natural products isolated from the brown alga *Cystoseira usneoides* [16,17] prompted us to evaluate the possible beneficial effects of AMT-E, one of the major active meroterpenoids of the alga, in an experimental model of colitis. The results have demonstrated that the treatment with this compound protected mice from the severity of DSS-induced colitis.

**Figure 7:**
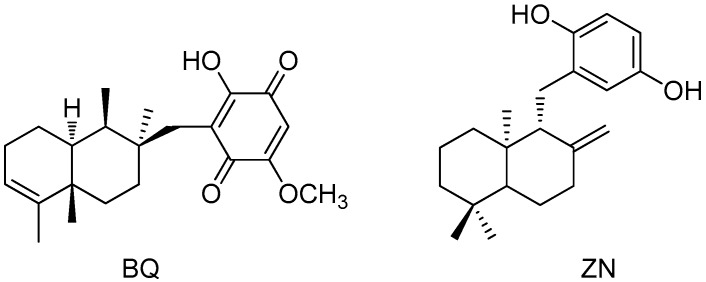
Chemical structures of marine meroterpenoids tested on inflammatory bowel disease (IBD) experimental models: bolinaquinone (BQ) [27] and zonarol (ZN) [28].

The DSS model mimics many of the features of human UC, such as diarrhoea, bloody faeces, body weight loss, mucosal ulceration, and shortening of colon length [22]. In the current study, based in an induction of damage by DSS for seven days and finished on day eight, oral administration of AMT-E (1, 10, and 20 mg/kg) significantly suppressed DSS-induced colitis, preventing body weight loss, colon shortening, and the extent of intestinal inflammation, although we did not observe rectal bleeding in any group assayed, nor differences in diarrheal stool between groups that received DSS (control and treated). In addition, microscopic analysis of the colon confirmed data from the macroscopic study, reflecting attenuation of the mucosal disruption and oedema after treatment with AMT-E. The in vivo anti-inflammatory effects of a few marine meroterpenoids, including avarol, avarone, and a series of polyprenylhydroquinones, have been previously demonstrated by using the TPA-induced ear edema or the carrageenan-induced paw edema experimental models [23,24,25,26]. More recently, the effects on experimental IBD have been described for bolinaquinone [27], a sesquiterpene quinone isolated from a sponge of the genus *Dysidea*, and zonarol [28], a sesquiterpene hydroquinone isolated from the brown alga *Dictyopteris undulata* (Figure 7). In particular, zonarol has been described to protect mice against DSS-induced UC via the inhibition of both inflammation and apoptosis. In agreement with our findings, zonarol-treated mice (20 mg/kg/day) also exhibited significantly suppressed DSS-induced colon shortening [28]. On the other hand, bolinaquinone (20 mg/kg/day) was found to protect mice against trinitrobenzene sulphonic acid (TNBS)-induced colonic inflammation [27].

Intestinal goblet cells produce the intestinal mucus, formed mainly by mucus glycoproteins or mucins. The intestinal mucus layer completely fills the crypts and acts both as a lubricant and as a physical barrier that protect the intestinal epithelial layer from injurious luminal stimulants [29]. Altered goblet cell physiology is considered as a hallmark of IBD pathology [4]. Further, clinical trials have indicated that IBD patients have decreased numbers of goblet cells and reduced mucus thickness [30]. The histological findings from our study revealed mucin-depleted crypts in mice with DSS-induced colitis, as attested by the loss of Alcian blue-stained goblet cells. Interestingly, treatments with AMT-E allows an accumulation of mucus inside goblet cells, which suggests a protective effect of this compound on the colonic epithelial damage that occurs in colitis. Our results are in line with those reported for bolinaquinone, which given at a dose of 20 mg/kg ameliorated the signs of TNBS-induced colitis, as assessed by histological examination of the colon [27]. However, the sesquiterpene hydroquinone zonarol did not significantly affect the number or size of mucus-producing goblet cells, as confirmed by Alcian blue or PAS staining [28].

In IBD, the disruption of the epithelial barrier implies the deregulation of the innate immune system by commensal flora, but also defects in the adaptive immune system [22]. One of the most prominent histological features observed in IBD is neutrophil infiltration into the inflamed mucosa and subsequently into the intestinal lumen, resulting in the formation of the so-called crypt abscesses [31]. During intestinal inflammation, neutrophils also contribute to the recruitment of other immune cells and facilitate mucosal healing by releasing inflammatory cytokines necessary for the resolution of inflammation [32]. In the present study, we have shown that MPO activity, an index of tissue-associated neutrophil accumulation, was significantly increased in the colonic mucosa after DSS administration. However, oral administration of AMT-E ameliorated polymorphonuclear infiltration into the colon, as evidenced by the suppression of colonic MPO activity as well as the improvement of histological features. These results suggest that inhibition of neutrophil accumulation by this meroterpene may be one of the protective mechanisms involved in reducing DSS-induced colonic mucosal injury.

To better understand the mechanism by which AMT-E ameliorates the DSS-induced colitis, we assessed the colonic production of the inflammatory cytokines TNF-α and IL-1β and the anti-inflammatory cytokine IL-10. Pro-inflammatory cytokines are known to play a pivotal role in the initiation and progression of the intestinal mucosa inflammation and immunity in IBD [33]. TNF-α may contribute to the induction of adhesion molecules and the expression of chemokines, resulting in an influx of inflammatory cells. Anti-TNF-α strategies are largely used in the clinical treatment of IBD [34], and the emerging data indicate that early use of anti-TNF-α antibodies leads to better long-term outcome in IBD patients by preventing mucosal damage [22,35]. The significance of increased IL-1β secretion in IBD has been also well-documented, and increased levels of IL-1β mRNA have been implicated in the production of many other inflammatory cytokines and also in the majority of IBD patients [36,37]. In the present study, the administration of AMT-E potently down-regulated the expression of these pro-inflammatory cytokines. Our results on the anti-inflammatory capacity of AMT-E are related to previous findings for zonarol, which has also been shown to inhibit the production of the pro-inflammatory cytokine TNF-α in DSS mice [28], and bolinaquinone, which was found to reduce the levels of IL-1β in the TNBS model of intestinal damage [27]. Concerning IL-10, this cytokine is an essential immune component in the intestinal tract, down-regulating the inflammatory process and helping to restore tissue homeostasis [38]. In animal models, a paper by Kuhn et al. [39] reported that IL-10-deficient mice developed chronic enterocolitis that can be prevented by administration of IL-10. Our group has also observed in this type of mice the spontaneous develop of chronic colitis and adenocarcinoma through a dysplasia sequence [40], and the reversal effects of experimental colitis by different treatments [41,42]. In the present study, the levels of IL-10 in the colon were found to be significantly increased in DSS mice and reduced in animals with higher doses, comparable to sham. This response suggests that the lower presence of pro-inflammatory cytokines by treatment do not trigger IL-10 production, and confirms less inflammation and lower hyperactive immune response.

Previous research on IBD has demonstrated that Th1/Th2 derived cytokines not only regulate their own synthesis, but also the expression of mediators and enzymes, including cyclooxygenase-2 (COX-2) and inducible nitric oxide synthase (iNOS) [5]. COX-2 and iNOS enzymes represent important molecular targets in IBD prevention and treatment. In active inflammation, cell destruction and increased permeability allow bacteria to enter the lamina propria. Inflammatory cytokines and bacterial antigens induce and drive the transcription of both COX-2 and iNOS. These two pro-inflammatory enzymes are also up-regulated in experimental colitis [42] and in active human IBD [43]. The expression and activity of these enzymes is associated with disease severity and involves them as potential anti-inflammatory drug targets. Our data clearly demonstrated that the colonic damage was associated with a higher expression of both COX-2 and iNOS proteins that were clearly reduced by AMT-E treatment. In line with our results, the marine meroterpenoid bolinaquinone has been reported to inhibit COX-2 and iNOS expression [27], while zonarol has only been shown to decrease the levels of iNOS expression in mice with DSS-induced UC [28].

In summary, our results have demonstrated for the first time that AMT-E, a natural product isolated from the alga *C. usneoides*, is effective in the protection against experimental colitis. The beneficial effects are mainly associated with macroscopic and microscopic data, and also with the markers of inflammation studied. Future studies are ensured, depending on the availability of AMT-E that will investigate deeper into the molecular mechanism by which AMT-E decreases intestinal inflammation as well as into the preventive ability of the compound in a nutraceutical approach, or the responses in chronic intestinal inflammation models. In any case, the set of effects allows us to assess the in vivo pharmacological properties of AMT-E, providing valuable information toward the development of new therapeutic molecules from macroalgae with potential benefits in inflammatory bowel disease.

## 4. Experimental Section

### 4.1. Experimental Animals

Seven-week-old female C57BL/6 mice weighing 18–20 g were purchased from Janvier Labs (France). Mice were housed on a regular 12 h light-dark cycle in a temperature (24–25 °C) and humidity (70%–75%) controlled room, and acclimated for 7 days. They were allowed free access to a laboratory diet (Panlab, Barcelona, Spain) and water ad libitum. The care and use of the animals and experimental protocol were approved by the Guidelines for the Animal Ethics Committee of the University of Seville, and all experiments in this study were carried out in accordance with the recommendations of the European Union regarding animal experimentation (Directive of the European Council 2010/63/EU).

### 4.2. Isolation of 11-Hydroxy-1′-O-Methylamentadione (AMT-E)

The meroditerpene AMT-E was isolated from the brown alga *Cystoseira usneoides* as previously described [17]. Briefly, the frozen alga was extracted with methanol, and after evaporation of the solution under reduced pressure, the aqueous residue was extracted with diethyl ether. The resulting extract was subjected to column chromatography (CC) on silica gel eluted with a mixture of *n*-hexane/diethyl ether (50:50, *v*/*v*), diethyl ether, chloroform/methanol mixtures (90:10 and 80:20, *v*/*v*), and finally methanol. The fractions eluted with diethyl ether and chloroform/methanol (90:10, *v*/*v*) were further separated by CC using as eluents *n*-hexane/ethyl acetate mixtures (80:20 to 30:70, *v*/*v*), ethyl acetate, and finally methanol. Selected fractions were subjected to repeated separations in normal phase HPLC using as eluent *n*-hexane/ethyl acetate (60:40, *v*/*v*) and in reversed phase HPLC using as eluent methanol/water (70:30, *v*/*v*). The isolated compound was identified from its ^1^H and ^13^C NMR spectra [16].

### 4.3. Induction of DSS Colitis and Treatments

Experimental colitis was induced by giving mice drinking water ad libitum containing 3% (*w*/*v*) DSS for 7 days. Mice of each of the groups were monitored carefully every day to confirm that they consumed an approximately equal volume of DSS-containing water. Animal body weights and water and food intake were recorded daily throughout all the experiments. On termination of the experiment on day eight, mice were killed by cervical dislocation. Colons were removed aseptically, slightly cleaned in physiological saline to remove faecal residues, weighed, and measured. Afterwards, the excised colons were cut longitudinally and small pieces from the middle to distal colon (areas of visible and inflammatory damage) were blotted dry, immediately frozen with liquid nitrogen, and stored at −80 °C until use.

Mice were divided randomly into five groups (twelve animals per group): (i) Sham group, which received drinking water, without DSS, throughout the experimental period (Sham); (ii) control group of induced colitis by DSS (DSS), and (iii–v) DSS groups treated with the compound AMT-E once a day during the 7 days at the doses of 1, 10 and 20 mg/ kg body weight and named AMT-E (1), AMT-E (10) and AMT-E (20), respectively. AMT-E was dissolved in vehicle consisting of 0.9% saline solution and 1% Tween-80 (Sigma-Chemical Co., St. Louis, MO, USA) and was administered by oral gavage. Both Sham and DSS groups received equal vehicle (0.9% saline solution/1% Tween-80) on the same schedule as AMT-E. All efforts were made to minimize the animals suffering and to reduce the number of animals used. The doses of AMT-E were chosen on the basis of literature with marine meroterpenoids in animal models of inflammation [23,27], including intestinal inflammation by DSS [28], and also in a short-preliminary experiment in our lab to acquire safety and efficacy in the DSS model of colitis. Criteria about potential translation of results from animal to humans were considered [44].

### 4.4. Histological Studies

For histological examination (4 animals per group), small sections (1 cm, approximately) from the middle to distal colon were excised and fixed in 4% paraformaldehyde in phosphate-buffered saline (PBS, pH 7.4), dehydrated by increasing concentrations of ethanol, and embedded in paraffin. Thereafter, sections of tissue were cut at 5 µm on a rotary microtome (Leica Microsystems, Wetzlar, Germany), mounted on clean glass slides and dried overnight at 37 °C. Sections were cleared, hydrated, and stained with haematoxylin and eosin, and Alcian blue for histological evaluation of colonic damage and mucus content, respectively, according to standard protocols; slides were coded to prevent observer bias during evaluation. All tissue sections were examined in an Olympus BH-2 microscope (GMI, Ramsey, MN, USA) for characterization of histopathology changes. The tissues were analysed by a blinded observer to establish a composite Histological Score as previously described [6], where researchers do not know the origin of the sample, except the coordinator. Criteria included mucosal architecture (0, absent; 1, mild; 2, medium; 3, severe), cellular infiltration (0, none; 1, infiltrate around the crypt basis; 2, infiltrate reaching the muscularis mucosae; 3, infiltrate reaching the submucosa), and goblet cell depletion (0, absent; 1, present). The results were expressed as the average scores of the 3 colonic sections.

### 4.5. Myeloperoxidase Activity Assay

Myeloperoxidase (MPO) is an enzyme found in neutrophils and, in much smaller quantities, in monocytes and macrophages. The MPO activity according to the method of Grisham et al. [45] was used as a convenient and valuable tool for evaluating neutrophil infiltration in the colon tissue. The colonic tissue samples were thawed, weighed, and homogenized in 10 volumes of 50 mM PBS, pH 7.4. The homogenate was centrifuged at 20,000× *g* for 20 min at 4 °C. The pellet was again homogenized in 10 volumes 50 mM PBS at pH 6.0, containing 0.5% hexadecyl trimethylammonium bromide (HETAB) and 10 mM EDTA. This homogenate was subjected to three cycles of freezing/thawing and a brief period of sonication. About 50 μL of homogenate sample was added to a 96-well microplate and incubated at 37 °C for 3 min with a mixture containing 0.067% *O*-dianisidine dihydrochloride, 0.5% HETAB, and 0.3 mM hydrogen peroxide. The changes in absorbance at 655 nm were measured with a microplate reader (Labysistem Multiskan EX, Helsinki, Finland). One unit of MPO activity was defined as the amount of enzyme present that produced a change in absorbance of 1.0 U/min at 37 °C in the final reaction volume containing the acetate. The results were expressed as U/mg tissue.

### 4.6. Cytokines Assay

Colonic samples for cytokine determinations (TNF-α, IL-1β, and IL-10) were weighed and homogenized at 4 °C after thawing, in a lysis buffer (1:5 *w*/*v*) containing PBS (pH 7.2), 1% bovine serum albumin (BSA), 0.01 mg/mL leupeptin, 0.01 mg/mL pepstatin, 0.01 mg/mL aprotinin, and 1 mM phenylmethylsulfonyl fluoride (PMSF). Then, the tubes were centrifugated at 12,000× *g* for 10 min at 4 °C; the supernatants were frozen at −80 °C until assay. Cytokines levels in frozen colonic tissue biopsy samples were measured with a commercially available ELISA kit (Diaclone, Besançon, France) according to the manufacturer’s instructions and then were expressed as nanograms per milligram of tissue.

### 4.7. Extraction of Cytoplasmic Proteins and Western Blot Analysis

Frozen colonic tissues were weighed and homogenized in ice-cold lysis buffer (50 mM Tris HCl, pH 7.5, 8 mM MgCl2, 5 mM ethylene glycol bis (2-aminoethyl ether)-*N*,*N*,*N′N′*-tetraacetic acid (EGTA), 0.5 mM EDTA, 0.01 mg/mL leupeptin, 0.01 mg/ml pepstatin, 0.01 mg/mL aprotinin, 1 mM PMSF, and 250 mM NaCl). Homogenates were incubated for 10 min on ice and centrifuged (12,000× *g* for 15 min at 4 °C), and the supernatants were collected and stored at −80°C. Protein concentration in the supernants was determined following colorimetric method of Bradford with gamma globulin as the standard [46]. Equal amounts of protein (50 μg) were then separated on 10% acrylamide gel by sodium dodecyl sulphate-polyacrylamide gel electrophoresis. In the next step, the proteins were electrophoretically transferred onto nitrocellulose membrane at 120 mA for 90 min. The membranes were then blocked in PBS-Tween 20 containing 5% *w*/*v* defatted milk. Later, the membranes were incubated with specific primary antibodies, rabbit anti-COX-2 and anti-iNOS (Cayman Chemical, Ann Arbour, MI, USA), at 4 °C overnight and 1:1000 dilution. To prove equal loading, the blots were analysed for β-actin expression using an anti-β-actin antibody (Sigma-Aldrich, St. Louis, MO, USA). Each membrane was washed three times for 15 min and incubated with the secondary horseradish peroxidase-linked anti-rabbit (Pierce Chemical, Rockford, IL, USA) for 60 min at room temperature. After washing the membranes again three times, the immunodetection was performed using an enhanced chemiluminescence light-detecting kit (Super-Signal West Pico Chemiluminescent Substrate, Pierce, IL, USA). Densitometric data were studied following normalization to the control (house-keeping gene). The signals were analysed and quantified with Scientific Imaging Systems (Biophotonics Image J Analysis Software).

### 4.8. Statistical Analysis

All data are expressed as arithmetic means ± standard error of the mean (S.E.M.) Data were evaluated with GraphPad Prism^®^ Version 5.00 software. The statistical significance of any difference in each parameter among the groups was evaluated by one-way analysis of variance (ANOVA) followed by Tukey test. *p* values of <0.05 were considered statistically significant. In the experiment involving histology, the figures shown are representative of at least three experiments performed on different days.

## Figures and Tables

**Table 1:** Effects of AMT-E (1, 10, and 20 mg/kg p.o.) on colonic length in dextran sodium sulphate (DSS)-treated mice.

Treatments (*n* = 12)	Sham ^a^	DSS ^b^	AMT-E ^c^ (1 mg/kg)	AMT-E ^c^ (10 mg/kg)	AMT-E ^c^ (20 mg/kg)
Colonic length (cm)	7.93 ± 0.16	6.02 ± 0.12 ^+++^	6.19 ± 0.10	7.00 ± 0.14	7.03 ± 0.16 **

**^a^** Sham group, receiving vehicle; **^b^** Group treated with DSS (3%) in drinking water, for 7 consecutive days; **^c^** Treated with DSS (3%) in drinking water and with AMT-E (1, 10, or 20 mg/kg, p.o.), for 7 consecutive days. Data are means ± S.E.M and were analysed by one-way ANOVA followed by Tukey test for comparison between groups. ^+++^ *p* < 0.001 vs. Sham group; ** *p* < 0.01 vs. DSS group.

**Table 2:** Effects of AMT-E on Histological Score in the dextran sodium sulphate (DSS) model.

Treatments	Sham	DSS	AMT-E (1 mg/kg)	AMT-E (10 mg/kg)	AMT-E (20 mg/kg)
**Colitis score**	0	3.93 ± 0.2 *	2.34 ± 0.1 ^†^	2.12 ± 0.2 ^†^	1.82 ± 0.2 ^†^

The criteria used for the histopathological scoring and immunostaining evaluation of colonic mucosa are described in the Experimental Section. Data are expressed as the means ± SEM. * *p* < 0.05 versus respective Sham group. ^†^ *p* < 0.05 versus DSS group.
